# The impact of clockwise and counterclockwise Le Fort I movements combined with bilateral sagittal split osteotomy on nasal morphology

**DOI:** 10.1007/s10006-026-01621-w

**Published:** 2026-08-19

**Authors:** Robin Hartmann, Marc Schoebel, Katharina Obermeier, Fabian Geyer, Maximilian Geyer, Wenko Smolka, Sven Otto, Waltraud Waiss, Tobias Ettl

**Affiliations:** 1 Department of Oral and Maxillofacial Surgery and Facial Plastic Surgery, LMU Medizin, LMU University Hospital, Ludwig-Maximilians-Universität München, Lindwurmstrasse 2a, 80337 Munich, Germany; 2 Department of Oral and Maxillofacial Surgery, University Hospital Regensburg, Franz-Josef-Strauß-Allee 11, 93053 Regensburg, Germany

**Keywords:** Le Fort I Osteotomy, Clockwise Rotation, Counterclockwise Rotation, Nasal Morphology, 3D Surface Imaging

## Abstract

**Purpose:**

Orthognathic surgery is known to induce measurable alterations in nasal morphology. This study retrospectively assessed pre- and postoperative three-dimensional (3D) surface models (SMs) to evaluate nasal soft-tissue changes associated with clockwise (CW) and counterclockwise (CCW) Le Fort I movements combined with bilateral sagittal split osteotomy (BSSO).

**Methods:**

Fifty-six patients (23 CW, 33 CCW) who underwent Le Fort I osteotomy with BSSO between January 2016 and February 2020 were included. 3D SMs were obtained 1 day preoperatively and 6 weeks postoperatively using the Vectra M5 stereophotogrammetry system (Canfield Scientific, USA). Seventeen anthropometric nasal parameters were measured with OnyxCeph3 (Image Instruments, Germany).

**Results:**

In the overall cohort, 8 of 17 nasal parameters showed significant postoperative changes. Most notably, transverse nasal dimensions increased, while nasal height and nasal dorsum length decreased. Subgroup analyses revealed that, after Holm–Bonferroni adjustment, 2 parameters remained significant in the CW group and 6 in the CCW group. However, direct between-group comparisons revealed no significant differences in the magnitude of postoperative changes.

**Conclusion:**

Le Fort I osteotomy combined with BSSO consistently alters nasal morphology, particularly by widening the alar and subalare regions and reducing nasal height. While both CW and CCW rotations produced measurable anthropometric changes, direct between-group comparisons showed no significant differences in the magnitude of postoperative changes.

## Introduction

Orthognathic surgery is indicated to correct functional disorders and improve facial aesthetics in cases of severe skeletal misalignments and jaw deformities [1, 2]. With advances in technology and surgical expertise, orthognathic surgery has become increasingly utilized, with German nationwide data showing a 61.8% rise in incidence between 2005 and 2022 [3]. Affecting both functional and aesthetic facial harmony, these procedures necessitate comprehensive outcome assessment, for which three-dimensional (3D) surface imaging provides a reliable and objective tool [4–7]. In particular, skeletal movements performed during orthognathic surgery may introduce consistent nasal soft-tissue changes. These measurable alterations in nasal and perioral morphology can be reliably assessed using 3D surface imaging [8]. Several authors have utilized 3D imaging for anthropometric assessments following orthognathic surgery [8–18]. In line with this, several studies specifically addressed nasal outcomes using 3D surface imaging after bimaxillary orthognathic surgery [19–21].

Despite the growing evidence, current studies remain heterogeneous in methodology, patient cohorts, and imaging systems, underscoring the need for further standardized investigations on nasal soft-tissue changes following orthognathic surgery using 3D surface imaging.

Therefore, this study aimed to retrospectively evaluate anthropometric nasal soft-tissue changes following bimaxillary surgery, consisting of Le Fort I osteotomy and bilateral sagittal split osteotomy (BSSO) according to Obwegeser/Dal Pont. Clockwise (CW) and counterclockwise (CCW) rotations of the maxilla were compared using 3D surface models (SMs) generated with the Vectra M5 stereophotogrammetry system (Canfield Scientific, USA) and a standardized set of anthropometric measurements analyzed with OnyxCeph3 software (Image Instruments, Germany).

## Materials and methods

### Study protocol

This retrospective single-center study was conducted at the Department of Oral and Maxillofacial Surgery, University Hospital Regensburg, Germany, following approval from the local ethics committee (25–4308-104). The investigation included 56 patients who underwent Le Fort I osteotomy and BSSO according to Obwegeser/Dal Pont at the Department of Oral and Maxillofacial Surgery at the University Hospital Regensburg between January 2016 and February 2020 [22, 23]. Preoperative planning was based on cone-beam computed tomography (CBCT), orthopantomograms (OPG), and lateral cephalometric radiographs. Virtual surgical planning was performed using OnyxCeph3 (Image Instruments, Chemnitz, Germany). Patients were allocated to the CW or CCW group according to the direction of maxillary pitch rotation defined in the virtual surgical plan generated with OnyxCeph3. Computer-aided design/computer-aided manufacturing (CAD/CAM) -manufactured intermediate and final occlusal splints were used intraoperatively. In addition, 3D facial surface images were acquired pre- and postoperatively using the Vectra M5 stereophotogrammetry system (Canfield Scientific, USA).

All patients underwent standardized Le Fort I osteotomy and BSSO according to Obwegeser/Dal Pont. An alar cinch suture was routinely performed in all patients at wound closure to minimize postoperative widening of the alar base.

### 3D surface imaging

The Vectra M5 stereophotogrammetry system was used to obtain 3D SMs 1 day preoperatively and approximately 6 weeks postoperatively. Stationary stereophotogrammetry systems are considered the gold standard for 3D surface imaging, providing an accuracy of approximately 1 mm compared with established reference methods, and their validity has been confirmed in several previous studies [24–26]. To ensure optimal and standardized image acquisition in 3D facial scanning, participants were seated with back support and instructed to maintain a natural head position, keep their lips relaxed and closed, and their eyes open consistently across sessions. Standard lighting conditions were employed to prevent artifacts caused by inconsistent illumination during image acquisition. SMs were exported in STL file format and imported into OnyxCeph3 for landmark identification and subsequent measurements. The software allows linear and angular measurements as well as three-dimensional analyses, and has been used in previous studies [27, 28]. Figures (Fig.) 1 and 2 illustrate the pre- and postoperative surface models of a representative patient following CW rotation surgery, while Figs. 3 and 4 depict the corresponding models after CCW rotation surgery.

**Fig. 1:**
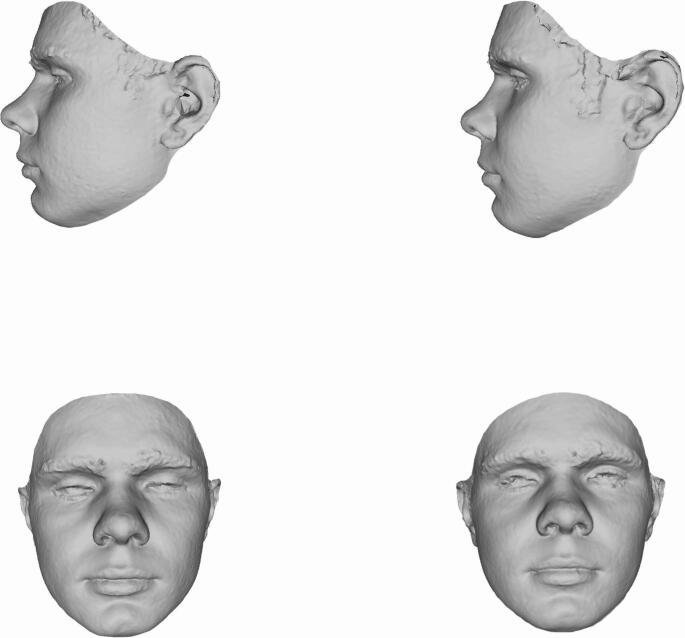
Appearance of a 19-year-old male patient before (left) and 6 weeks after (right) CW Le Fort I osteotomy combined with BSSO

**Fig. 2:**
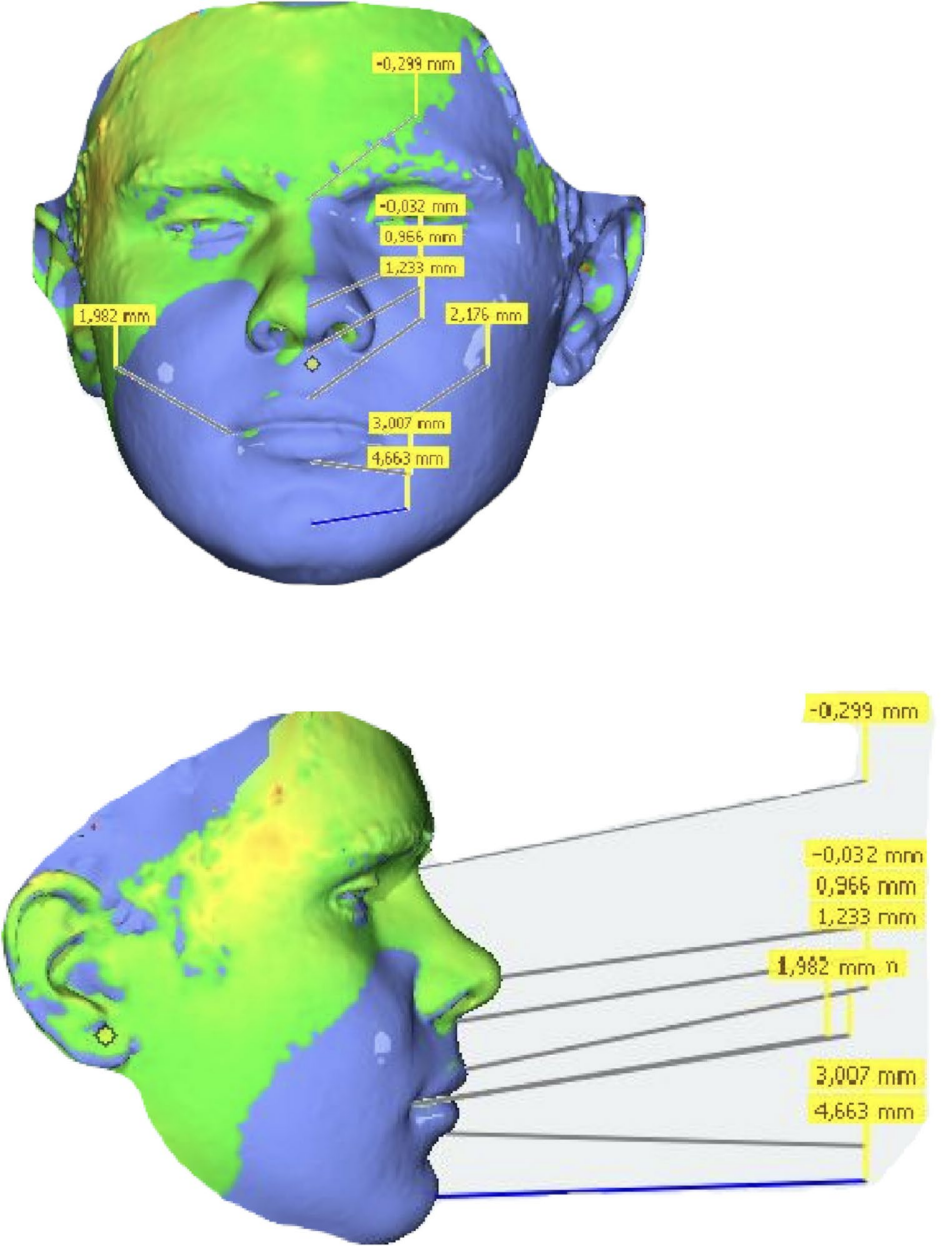
Color-coded surface superimposition of the pre- and postoperative three-dimensional surface models of the same 19-year-old male patient shown in Fig. 1, following CW Le Fort I osteotomy combined with BSSO, displayed in frontal (top) and lateral (bottom) view. The postoperative model was registered to the preoperative model, and the closest-point distance between both surfaces was mapped onto the face. Numerical labels indicate the local pre- to postoperative surface displacement (mm) at selected landmarks

**Fig. 3:**
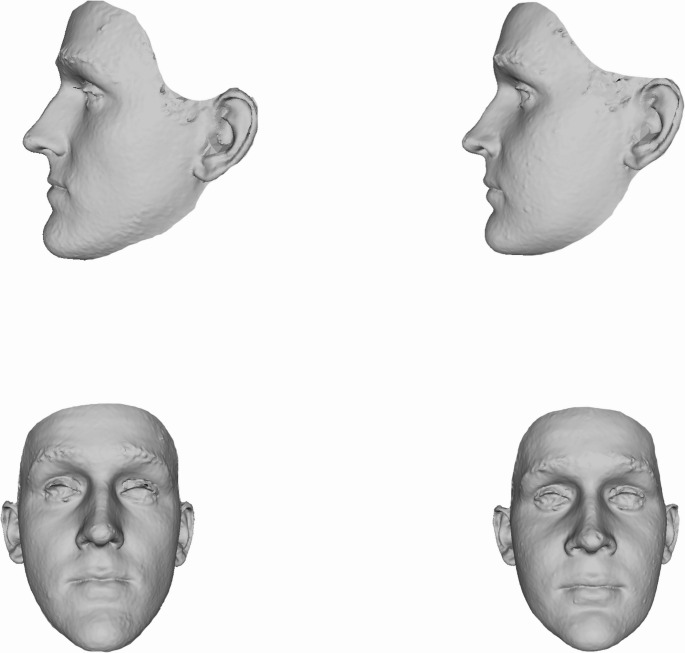
Appearance of a 23-year-old male patient before (left) and 6 weeks after (right) CCW Le Fort I osteotomy combined with BSSO

**Fig. 4:**
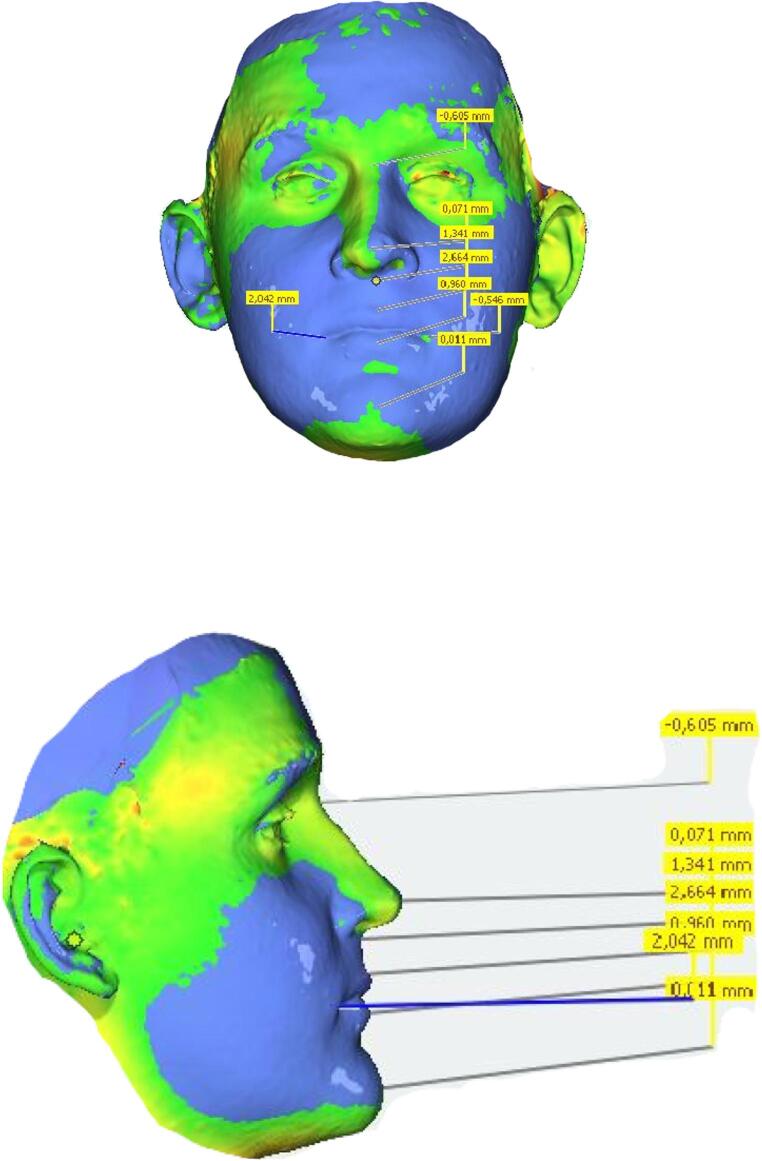
Color-coded surface superimposition of the pre- and postoperative three-dimensional surface models of the same 23-year-old male patient shown in Fig. 3, following CCW Le Fort I osteotomy combined with bilateral sagittal split osteotomy (BSSO), displayed in frontal (top) and lateral (bottom) view. Registration, color mapping and numerical labeling as described for Fig. 2

### Landmarks

**Fig. 5:**
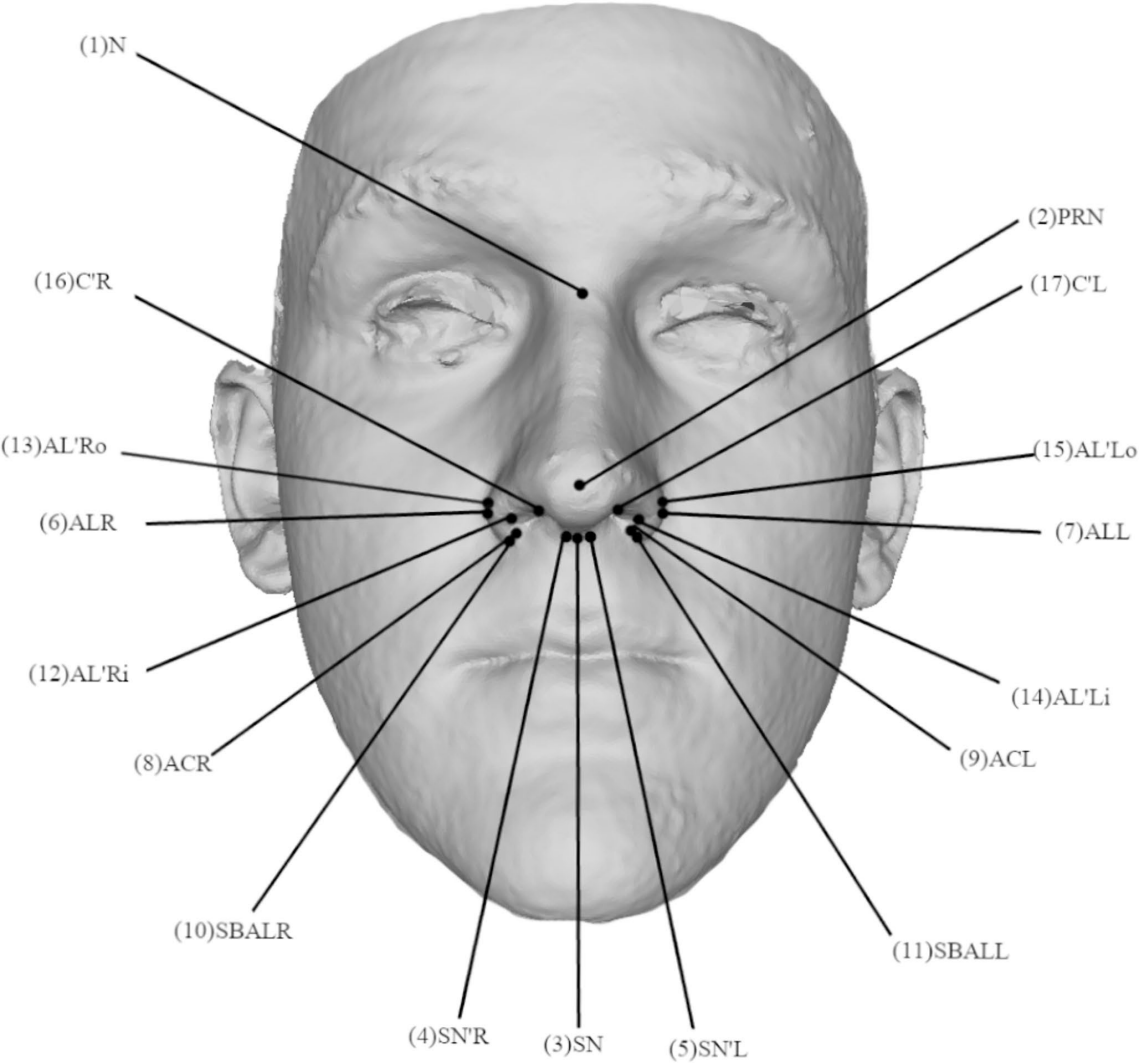
Anthropometric landmarks used for nasal soft-tissue analysis. (1) N (Nasion), (2) PRN (Pronasale), (3) SN (Subnasale), (4) SN′R (midpoint of the columellar base, right), (5) SN′L (midpoint of the columellar base, left), (6) ALR (Alare right), (7) ALL (Alare left), (8) ACR (Alar curvature right), (9) ACL (Alar curvature left), (10) SBALR (Subalare right), (11) SBALL (Subalare left), (12) AL′Ri (inner reference point for nasal wing thickness, right), (13) AL′Ro (outer reference point for nasal wing thickness, right), (14) AL′Li (inner reference point for nasal wing thickness, left), (15) AL′Lo (outer reference point for nasal wing thickness, left), (16) C′R (highest point of the right columella), (17) C′L (highest point of the left columella)

Standard landmark sets were semi-automatically detected by the OnyxCeph3 software. Each proposed landmark was subsequently reviewed and confirmed by a trained observer to ensure accuracy and consistency. The following 17 landmarks were defined according to the OnyxCeph3 protocol: (1) N (Nasion), (2) PRN (Pronasale), (3) SN (Subnasale), (4) SN′R (midpoint of the columellar base, right), (5) SN′L (midpoint of the columellar base, left), (6) ALR (Alare right), (7) ALL (Alare left), (8) ACR (Alar curvature right), (9) ACL (Alar curvature left), (10) SBALR (Subalare right), (11) SBALL (Subalare left), (12) AL′Ri (inner reference point for nasal wing thickness, right), (13) AL′Ro (outer reference point for nasal wing thickness, right), (14) AL′Li (inner reference point for nasal wing thickness, left), (15) AL′Lo (outer reference point for nasal wing thickness, left), (16) C′R (highest point of the right columella), (17) C′L (highest point of the left columella). Figure 5 shows the landmarks used for anthropometric assessment.

### Anthropometric measurements

**Fig. 6:**
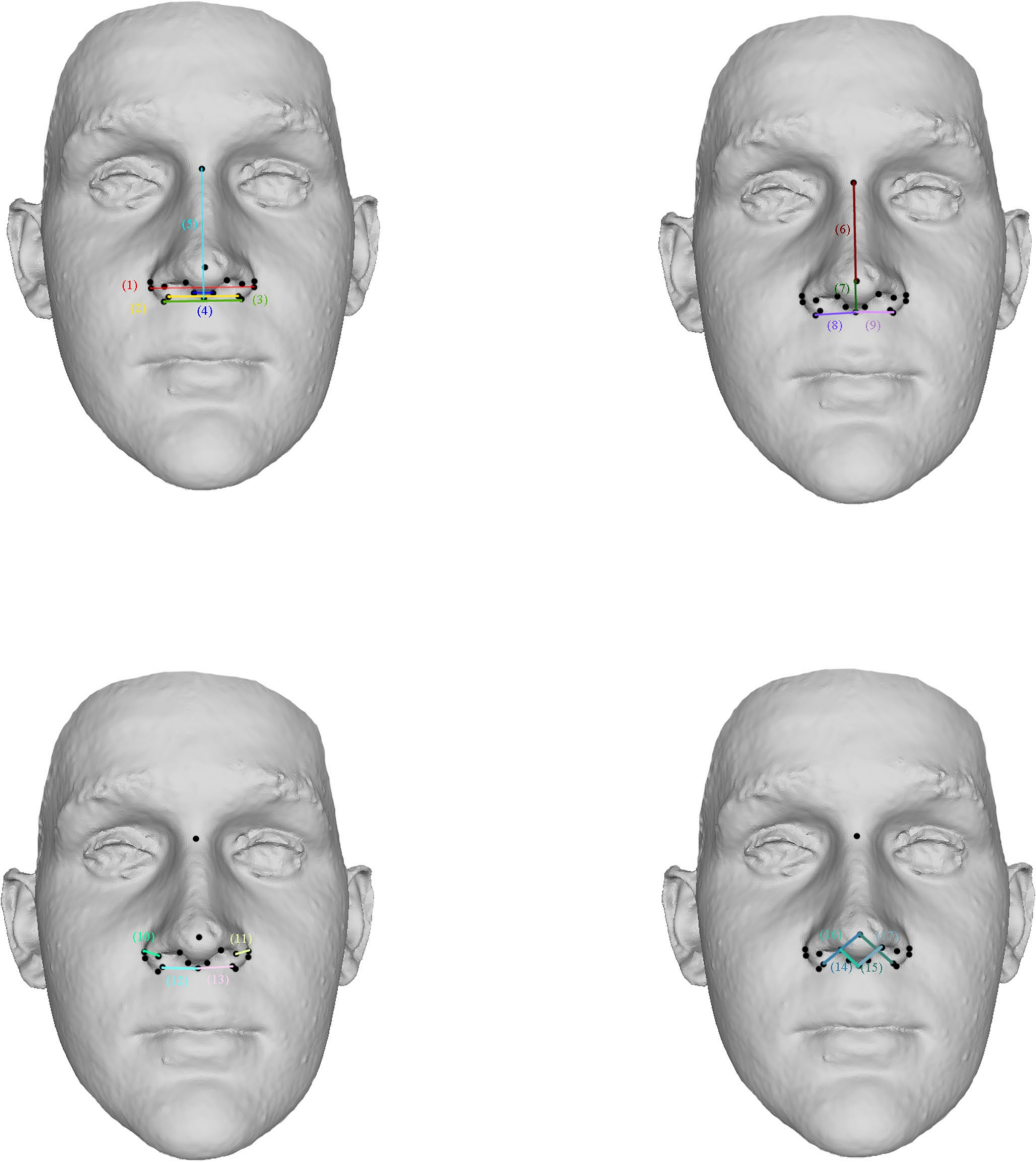
Anthropometric nasal measurements performed in this study: (1) ALR–ALL (lower nasal width), (2) ACR–ACL (alar base width), (3) SBALL–SBALR (width between subalare points), (4) SN′L–SN′R (nasal septum width), (5) N–SN (nasal height), (6) N–PRN (nasal dorsum length), (7) SN–PRN (nasal tip projection), (8) SBALR–SN (nasal base width right), (9) SN–SBALL (nasal base width left), (10) AL′Ri–AL′Ro (nasal wing thickness right), (11) AL′Li–AL′Lo (nasal wing thickness left), (12) ACR–SN (alar base to subnasale distance right), (13) SN–ACL (subnasale to alar base distance left), (14) ACR–PRN (nasal wing length right), (15) PRN–ACL (nasal wing length left), (16) SN–C′R (columella length right), (17) SN–C′L (columella length left)

The following measurements were performed: (1) ALR–ALL (lower nasal width), (2) ACR–ACL (alar base width), (3) SBALL–SBALR (width between subalare points), (4) SN′L–SN′R (nasal septum width), (5) N–SN (nasal height), (6) N–PRN (nasal dorsum length), (7) SN–PRN (nasal tip projection), (8) SBALR–SN (nasal base width right), (9) SN–SBALL (nasal base width left), (10) AL′Ri – AL′Ro (nasal wing thickness right), (11) AL′Li – AL′Lo (nasal wing thickness left), (12) ACR–SN (alar base to subnasale distance right), (13) SN–ACL (subnasale to alar base distance left), (14) ACR–PRN (nasal wing length right), (15) PRN–ACL (nasal wing length left), (16) SN–C′R (columella length right), (17) SN–C′L (columella length left). Figure 6 gives an overview of all anthropometric measurements performed in this investigation.

All scans and anthropometric measurements were carried out by a single experienced healthcare professional. To minimize systematic bias, all pre- and postoperative scans were analyzed using identical landmark definitions and a software-assisted detection protocol (OnyxCeph3).

### Statistical analysis

Statistical analyses were performed using IBM SPSS Statistics version 29.0 (IBM Corp., USA). Normality of data distribution was assessed using the Kolmogorov–Smirnov test. Descriptive statistics were calculated, presenting continuous variables as mean ± standard deviation (SD) and categorical variables as counts and percentages.

Group differences in demographic and clinical characteristics (age, sex, dysgnathic class) were analyzed using the Mann–Whitney U test for continuous variables and the chi-square test for categorical variables. Pre- to postoperative comparisons of anthropometric nasal measurements within the total sample and within the CW and CCW subgroups were performed using paired-samples t-tests. If the assumption of normality was not met, the Wilcoxon signed-rank test was applied.

A p-value < 0.05 was considered statistically significant. To account for the increased risk of type I error due to multiple comparisons, adjustment of the significance level was carried out using the Holm–Bonferroni method. The α-level adjustment was calculated using the online tool described by Hemmerich et al. [29]. Interpretation of statistical significance was based on Holm–Bonferroni–adjusted p-values, unadjusted p-values are reported for transparency.

## Results

### Patient demographics

A total of 56 patients were included in the study, comprising 23 patients in the CW rotation group and 33 patients in the CCW rotation group. The mean age of the entire cohort was 24.0 ± 6.4 years, with no significant difference between groups (*p* = 0.973). Overall, 42.9% of the patients were female (39.1% in the CW group and 45.5% in the CCW group; *p* = 0.638). Dysgnathic class II was present in 41.1% of patients (47.8% CW, 36.4% CCW), while 58.9% exhibited class III dysgnathia (52.2% CW, 63.6% CCW; *p* = 0.391). The mean rotational movement of the maxilla was 3.20 ± 2.58° in the CW group and − 2.18 ± 1.55° in the CCW group, yielding an overall mean of 0.03 ± 3.35° Table (Tab.) 1.

**Table 1:** Demographic and clinical characteristics of the study groups: Values are presented as mean ± standard deviation (SD) for continuous variables and as absolute numbers with percentages [n (%)] for categorical variables. P-values were obtained using the Mann–Whitney U test for age, and the chi-square test for categorical variables

	Demographic Data				
Variables		**Clockwise (n = 23)**	**Counter-clockwise (n = 33)**	**Overall (n = 56)**	
		Mean ± SD/n (%)	Mean ± SD/n (%)	Mean ± SD/n (%)	p-value
Age (years)		24.0 ± 6.4	24.0 ± 6.4	24.0 ± 6.4	0.973
Sex, female		9 (39.1%)	15 (45.5%)	24 (42.9%)	0.638
Sex, male		14 (60.9%)	18 (54.5%)	32 (57.1%)	
Dysgnathic class 2		11 (47.8%)	12 (36.4%)	23 (41.1%)	0.391
Dysgnathic class 3		12 (52.2%)	21 (63.6%)	33 (58.9%)	
Rotation (degree)		3.20 ± 2.58	−2.18 ± 1.55	0.03 ± 3.35	

### Overall nasal soft-tissue changes

Overall nasal soft-tissue changes are presented in Table 2.

**Table 2:** Comparison of anthropometric measurements before and after orthognathic surgery (total sample). Values are presented as mean ± standard deviation (SD). Differences represent post- minus preoperative values. Anthropometric measurements are reported in millimeters (mm). P-values were primarily obtained using paired-samples t-tests. For measurements (7), (8), and (10), significance values were derived from the Wilcoxon signed-rank test due to non-normal data distribution. Both unadjusted and Holm–Bonferroni–adjusted p-values are reported. Data analysis was performed using IBM SPSS Statistics 29

	Comparison of Anthropometric Measurements before and after Orthognathic Surgery (Total Sample) (*N* = 56) Total sample (*N* = 56)											
Variables		Pre-OP			Post-OP			Difference				
		Mean	SD	Mean		SD	Mean		SD	p		p adj.
(1) ALR–ALL		34.7	2.9	37.2		2.8	2.5		1.8	< 0.001		0.017*
(2) ACR–ACL		28.7	3.5	32.0		3.1	3.2		3.0	< 0.001		0.017*
(3) SBALL–SBALR		18.7	3.2	20.0		2.7	1.3		2.6	< 0.001		0.017*
(4) SN′L–SN′R		11.3	2.1	11.3		2.5	0.0		2.4	0.970		> 0.999
(5) N–SN		51.4	3.9	50.5		3.9	− 0.8		1.6	< 0.001		0.017*
(6) N–PRN		45.5	4.4	44.8		4.3	− 0.7		1.7	0.003		0.033*
(7) SN–PRN		19.5	1.9	19.0		1.6	− 0.5		1.4	0.025		0.225
(8) SBALR–SN		11.7	1.8	11.7		1.6	0.1		1.7	0.311		> 0.999
(9) SN–SBALL		11.5	2.1	11.8		1.8	0.3		1.5	0.143		0.715
(10) AL′Ri–AL′Ro		7.3	1.1	7.1		1.2	− 0.1		1.3	0.873		> 0.999
(11) AL′Li–AL′Lo		7.1	1.0	7.3		1.3	0.2		1.4	0.316		> 0.999
(12) ACR–SN		17.4	2.3	18.1		1.9	0.7		1.8	0.004		0.040*
(13) SN–ACL		17.2	2.4	17.8		2.1	0.6		2.0	0.033		0.264
(14) ACR–PRN		29.9	2.4	29.1		2.1	− 0.7		1.4	< 0.001		0.017*
(15) PRN–ACL		30.2	2.6	29.3		2.3	− 0.8		1.7	< 0.001		0.017*
(16) SN–C′R		12.2	1.7	11.8		1.5	− 0.3		1.6	0.117		0.702
(17) SN–C′L		11.8	1.6	11.4		1.6	− 0.4		1.4	0.051		0.357

When comparing pre- to postoperative measurements in the total sample (*N* = 56), 8 out of 17 nasal parameters showed statistically significant differences after Holm–Bonferroni adjustment. Significant widening was observed for (1) ALR–ALL (lower nasal width; Δ = +2.5 ± 1.8 mm, adjusted *p* = 0.017), (2) ACR–ACL (alar base width; Δ = +3.2 ± 3.0 mm, adjusted *p* = 0.017), and (3) SBALL–SBALR (width between subalare points; Δ = +1.3 ± 2.6 mm, adjusted *p* = 0.017). In contrast, (5) N–SN (nasal height; Δ = −0.8 ± 1.6 mm, adjusted *p* = 0.017) and (6) N–PRN (nasal dorsum length; Δ = −0.7 ± 1.7 mm, adjusted *p* = 0.033) significantly decreased. Further significant reductions were noted for (14) ACR–PRN (nasal wing length right; Δ = −0.7 ± 1.4 mm, adjusted *p* = 0.017) and (15) PRN–ACL (nasal wing length left; Δ = −0.8 ± 1.7 mm, adjusted *p* = 0.017). A significant increase was observed for (12) ACR–SN (alar base to subnasale distance right; Δ = +0.7 ± 1.8 mm, adjusted *p* = 0.040) (Table 2).

No significant differences were found for (4) septal width (SN′L–SN′R; adjusted *p* > 0.999), (8, 9) right and left subnasale–subalare distances (adjusted *p* > 0.999 and *p* = 0.715), (10, 11) alar thickness on both sides (adjusted *p* > 0.999 and *p* > 0.999), (13) subnasale to alar base distance left (SN–ACL; adjusted *p* = 0.264), (7) nasal tip projection (SN–PRN; adjusted *p* = 0.225), and (16, 17) columella length on the right and left side (SN–C′R, adjusted *p* = 0.702; SN–C′L, adjusted *p* = 0.357) (Table 2).

### Clockwise group

In the CW group, 2 of the 17 nasal parameters demonstrated statistically significant postoperative changes after Holm–Bonferroni adjustment (Table 3). Significant widening was observed for (1) ALR–ALL (lower nasal width; Δ = +2.3 ± 1.8 mm, adjusted *p* = 0.017) and (2) ACR–ACL (alar base width; Δ = +2.9 ± 3.2 mm, adjusted *p* = 0.017). None of the remaining parameters showed statistically significant postoperative changes after adjustment for multiple comparisons (Table 3).

**Table 3:** Comparison of anthropometric measurements before and after orthognathic surgery in the Clockwise Group. Values are presented as mean ± standard deviation (SD). Anthropometric measurements are reported in millimeters (mm). Differences represent post- minus preoperative values. P-values were primarily obtained using paired-samples t-tests. For measurement (7) SN–PRN, significance values were derived from the Wilcoxon signed-rank test due to non-normal data distribution. Both unadjusted and Holm–Bonferroni–adjusted p-values are reported. Data analysis was performed using IBM SPSS Statistics 29

	Comparison of Anthropometric Measurements before and after Orthognathic Surgery (Clockwise Group) (*N* = 23)												
Variables		Pre-OP				Post-OP			Difference				
		Mean		SD	Mean		SD	Mean		SD	p		p adj.
(1) ALR–ALL			35.5	2.67	37.8		2.07	2.3		1.83	< 0.001		0.017*
(2) ACR–ACL			29.4	3.50	32.3		3.17	2.9		3.17	< 0.001		0.017*
(3) SBALL–SBALR			19.4	2.80	20.5		3.17	1.1		2.55	0.059		0.767
(4) SN′L–SN′R			12.4	2.01	11.5		2.68	− 0.8		2.95	0.186		> 0.999
(5) N–SN			51.7	4.67	50.9		4.42	− 0.8		2.00	0.066		0.792
(6) N–PRN			45.8	5.45	45.0		5.06	− 0.8		1.73	0.036		0.504
(7) SN–PRN			19.6	1.87	19.6		1.43	0.0		1.20	0.726		> 0.999
(8) SBALR–SN			12.1	2.10	11.8		1.88	− 0.3		1.82	0.388		> 0.999
(9) SN–SBALL			12.0	2.27	12.3		2.24	0.3		1.52	0.335		> 0.999
(10) AL′Ri–AL′Ro			7.9	1.09	7.6		1.30	− 0.3		1.30	0.287		> 0.999
(11) AL′Li–AL′Lo			7.5	0.94	7.7		1.28	0.2		1.31	0.473		> 0.999
(12) ACR–SN			18.1	2.38	18.5		1.89	0.4		2.09	0.343		> 0.999
(13) SN–ACL			17.9	2.52	18.1		2.62	0.2		2.32	0.676		> 0.999
(14) ACR–PRN			30.5	2.59	29.9		2.28	− 0.6		1.69	0.117		> 0.999
(15) PRN–ACL			31.0	2.60	30.1		2.40	− 0.9		1.82	0.025		0.375
(16) SN–C′R			12.0	1.71	12.0		1.35	0.0		1.42	0.896		> 0.999
(17) SN–C′L			11.7	1.51	11.7		1.52	0.0		1.37	0.880		> 0.999

### Counterclockwise group

In the CCW group, 6 of the 17 nasal parameters demonstrated statistically significant postoperative changes after Holm–Bonferroni adjustment (Table 4). Significant widening was observed for (1) ALR–ALL (lower nasal width; Δ = +2.7 ± 1.8 mm, adjusted *p* = 0.017) and (2) ACR–ACL (alar base width; Δ = +3.5 ± 2.9 mm, adjusted *p* = 0.017). In addition, (12) ACR–SN (alar base to subnasale distance right) increased significantly (Δ = +1.0 ± 1.6 mm, adjusted *p* = 0.026). Significant reductions were observed for (5) N–SN (nasal height; Δ = −0.9 ± 1.4 mm, adjusted *p* = 0.017), (14) ACR–PRN (nasal wing length right; Δ = −0.9 ± 1.1 mm, adjusted *p* = 0.017), and (7) SN–PRN (nasal tip projection; Δ = −0.8 ± 1.4 mm, adjusted *p* = 0.036). None of the remaining parameters showed statistically significant postoperative changes after adjustment for multiple comparisons (Table 4).

**Table 4:** Comparison of anthropometric measurements before and after orthognathic surgery in the Counterclockwise group. Values are presented as mean ± standard deviation (SD). Differences represent post- minus preoperative values. Anthropometric measurements are reported in millimeters (mm). P-values were primarily obtained using paired-samples t-tests. For measurement (10) AL′Ri–AL′Ro, significance values were derived from the Wilcoxon signed-rank test. Both unadjusted and Holm–Bonferroni–adjusted p-values are reported. Data analysis was performed using IBM SPSS Statistics 29

	Comparison of Anthropometric Measurements before and after Orthognathic Surgery (Counterclockwise Group) (*N* = 33)											
Variables		Pre-OP			Post-OP			Difference				
		Mean	SD	Mean		SD	Mean		SD	p		p adj.
(1) ALR–ALL		34.1	2.98	36.8		3.16	2.7		1.76	< 0.001		0.017*
(2) ACR–ACL		28.3	3.53	31.7		3.01	3.5		2.93	< 0.001		0.017*
(3) SBALL–SBALR		18.2	3.36	19.6		2.29	1.4		2.74	0.006		0.066
(4) SN′L–SN′R		10.5	1.76	11.1		2.38	0.6		1.87	0.093		0.465
(5) N–SN		51.1	3.36	50.3		3.46	− 0.9		1.38	0.001		0.017*
(6) N–PRN		45.2	3.63	44.6		3.74	− 0.6		1.64	0.044		0.308
(7) SN–PRN		19.5	1.88	18.7		1.62	− 0.8		1.38	0.003		0.036*
(8) SBALR–SN		11.3	1.57	11.7		1.50	0.3		1.51	0.223		0.892
(9) SN–SBALL		11.1	1.92	11.4		1.34	0.3		1.57	0.281		0.892
(10) AL′Ri–AL′Ro		6.8	0.93	6.8		1.07	0.0		1.27	0.645		0.974
(11) AL′Li–AL′Lo		6.8	0.88	7.0		1.20	0.2		1.41	0.487		0.974
(12) ACR–SN		16.9	2.08	17.9		1.94	1.0		1.64	0.002		0.026*
(13) SN–ACL		16.8	2.31	17.6		1.75	0.9		1.78	0.009		0.090
(14) ACR–PRN		29.5	2.28	28.6		1.86	− 0.9		1.12	< 0.001		0.017*
(15) PRN–ACL		29.6	2.45	28.9		2.07	− 0.8		1.67	0.011		0.099
(16) SN–C′R		12.3	1.73	11.7		1.60	− 0.6		1.63	0.044		0.308
(17) SN–C′L		11.8	1.61	11.2		1.62	− 0.6		1.42	0.019		0.152

### Comparison of clockwise vs. counterclockwise groups

Direct comparison of postoperative changes between the CW (*N* = 23) and CCW (*N* = 33) groups revealed that the magnitude of postoperative changes did not differ significantly between the two surgical movements for all nasal parameters (Table 5).

**Table 5:** Comparison of pre- to postoperative anthropometric changes between clockwise and counterclockwise rotation groups

		Comparison of Pre- to Postoperative Anthropometric Changes Between Clockwise and Counterclockwise Rotation Groups								
Variables			ΔClockwise group			ΔCounterclockwise group				
			Mean	SD	Mean		SD	p	p adj.	
(1) ΔALR–ALL			2.3	1.83	2.7		1.76	0.478	> 0.999	
(2) ΔACR–ACL			2.9	3.17	3.5		2.93	0.506	> 0.999	
(3) ΔSBALL–SBALR			1.1	2.55	1.4		2.74	0.627	> 0.999	
(4) ΔSN′L–SN′R			− 0.8	2.95	0.6		1.87	0.034	0.544	
(5) ΔN–SN			− 0.8	2.00	− 0.9		1.38	0.898	> 0.999	
(6) ΔN–PRN			− 0.8	1.73	− 0.6		1.64	0.650	> 0.999	
(7) ΔSN–PRN			0.0	1.20	− 0.8		1.38	0.028	0.476	
(8) ΔSBALR–SN			− 0.3	1.82	0.3		1.51	0.072	> 0.999	
(9) ΔSN–SBALL			0.3	1.52	0.3		1.57	0.975	> 0.999	
(10) ΔAL′Ri–AL′Ro			− 0.3	1.30	0.0		1.27	0.453	> 0.999	
(11) ΔAL′Li–AL′Lo			0.2	1.31	0.2		1.41	0.942	> 0.999	
(12) ΔACR–SN			0.4	2.09	1.0		1.64	0.284	> 0.999	
(13) ΔSN–ACL			0.2	2.32	0.9		1.78	0.236	> 0.999	
(14) ΔACR–PRN			− 0.6	1.69	− 0.9		1.12	0.467	> 0.999	
(15) ΔPRN–ACL			− 0.9	1.82	− 0.8		1.67	0.784	> 0.999	
(16) ΔSN–C′R			0.0	1.42	− 0.6		1.63	0.139	> 0.999	
(17) ΔSN–C′L			0.0	1.37	− 0.6		1.42	0.142	> 0.999	

Comparison of pre- to postoperative changes (Δ) in anthropometric nasal measurements between the clockwise (CW) and counterclockwise (CCW) rotation groups. Values are presented as mean ± standard deviation (SD). Differences represent postoperative minus preoperative values in millimeters (mm). P-values were primarily obtained using independent-samples t-tests. For measurements (4) ΔSN′L–SN′R, (7) ΔSN–PRN, and (10) ΔAL′Ri–AL′Ro, significance values were derived from the Mann–Whitney U test due to non-normal data distribution. Both unadjusted and Holm–Bonferroni–adjusted p-values are reported. Data analysis was performed using IBM SPSS Statistics 29

## Discussion

Nasal soft-tissue changes after orthognathic surgery have been examined in several previous studies using different 3D surface imaging systems. Worasakwutiphong et al. analyzed pre- to postoperative nasal soft-tissue measurements in 38 patients with Class III malocclusion undergoing bimaxillary orthognathic surgery, using the 3dMD system (3dMD Inc, USA) [19]. Chen et al. performed a retrospective cohort study comparing nasal soft-tissue changes after CW and CCW bimaxillary orthognathic surgery in 37 Asian Class III patients [20]. Shi et al. evaluated anthropometric soft-tissue changes, including nasolabial dimensions, in 30 skeletal Class III patients undergoing bimaxillary orthognathic surgery, using the Artec EVA structured light scanner (Artec 3D, Luxembourg) [21]. DeSesa et al. conducted a retrospective cohort study on nasolabial soft-tissue changes following different Le Fort I subtypes in 108 three-dimensional photogrammetric datasets acquired with the VECTRA system (Canfield Scientific, USA) [18].

In the present study transverse widening (ALR–ALL, ACR–ACL, SBALL–SBALR) was accompanied by reductions in nasal height and nasal dorsum length. These findings align with earlier reports. Worasakwutiphong et al. also observed significant alterations in nasolabial morphology after bimaxillary surgery, namely an increase in alar width (+ 0.74 mm), enlargement of the nasolabial angle (+ 9.2°), and a reduction of tip projection (–1.99 mm) [19]. However, in contrast to the present study, they reported relative stability of transverse dimensions (width of the bilateral alar base and subalare). In line with our results, DeSesa et al. found significant nasal changes, too, particularly an increase of alar base width, alar width and nostril width with Le Fort I advancement [18]. Alar cinch sutures are suggested to reduce that problem. However, the extent of transversal narrowing may pose some difficulties. In our daily practice the paranasal muscles are readapted with a slight medialization. Nasal tip projection decreased significantly in the CCW group after Holm–Bonferroni adjustment (Δ = −0.8 ± 1.4 mm, adjusted *p* = 0.036), whereas no significant change was observed in the CW group or in the overall cohort. The direct between-group comparison of these changes did not reach significance after adjustment (adjusted *p* = 0.476), and this dissociation should therefore not be interpreted as evidence of a true difference between rotational directions.

The magnitude of changes remained comparable between rotational directions. This is in line with Chen et al., who reported similar postoperative alar widening in CW and CCW groups, suggesting that pitch direction may not be the primary determinant of nasal widening [20].

The observed widening, however, aligns with findings from previous investigations. Chen et al. also found increases in alar width following both CW and CCW rotations (CW + 1.04 mm; CCW + 1.22 mm) [20]. Shi et al., employing structured light scanning (Artec EVA), emphasized nasolabial angle changes with columella shortening alongside an increase in nasal base width (+ 1.38 mm) [21]. While the present study also showed a descriptive reduction in columella length (SN–C′R, Δ = −0.3 ± 1.6 mm; SN–C′L, Δ = −0.4 ± 1.4 mm) and an increase in nasal base width, the reduction in columella length did not reach statistical significance after Holm–Bonferroni adjustment, and the magnitude of changes differed. One explanation may be methodological. While the present study employed semi-automatic landmarking within OnyxCeph3, Shi et al. utilized CMF ProPlan 3.0 software, which may differ in its ability to detect sagittal and vertical alterations. These aspects highlight the importance of standardized imaging and surgical protocols for comparability of nasal soft-tissue outcomes after orthognathic surgery. As a retrospective study, surface measurements were assessed by a single observer, which may limit generalizability of this study’s findings. Future investigations should include assessments of both inter- and intra-rater reliability to strengthen the validity of the findings.

A limitation of the present study is the relatively short postoperative follow-up period of six weeks. While this time point was chosen as substantial postoperative swelling is expected to have subsided by this time point, although residual edema and ongoing soft-tissue remodeling cannot be excluded, it may not fully reflect the long-term stability of nasal soft-tissue changes after orthognathic surgery. Therefore, the findings should be interpreted with this limitation in mind. Future studies with longer follow-up periods are needed to determine whether the observed changes remain stable over time.

A further limitation of this study relates to the assessment of skeletal movements. Since the primary aim was to evaluate soft-tissue alterations of the nose using three-dimensional SMs, the study did not quantify differences in maxillary impaction and sagittal translation between the operated groups beyond the mean rotational movement of the maxilla, nor the accuracy of surgical execution. Future studies could combine a detailed analysis of skeletal movements with three-dimensional soft-tissue assessment of anthropometric changes in nasal morphology.

In addition, the process of identifying landmarks warrants discussion. In this study, landmarks were not pre-marked prior to surface imaging, which may introduce measurement errors. Previous investigations have used colored stickers or eyeliner to facilitate landmark identification during anthropometric assessments, thereby potentially improving accuracy [30–33]. However, in the present trial this was not deemed necessary, as the semi-automatic landmark detection implemented by the software allowed for precise identification without pre-scan marking. Concordantly, some studies even recommend omitting pre-scan landmarking. In their investigation Aynechi et al. demonstrated that accurate and precise measurements can be obtained without prior manual landmark identification [34].

Overall, the present study’s findings confirm previous reports regarding nasal soft tissue changes after orthognathic surgery. However, by standardizing protocols and imaging techniques, the reliability and comparability of such methods may be further improved. 3D surface imaging allows objective quantification of these nasal soft-tissue changes and may help surgeons anticipate them during surgical planning and patient counseling.

## Conclusion

Bimaxillary orthognathic surgery was associated with consistent and measurable alterations of nasal soft-tissue morphology. In the overall cohort, 8 of 17 nasal parameters demonstrated significant postoperative changes, most notably a widening of the alar base and subalare region, together with reductions in nasal height and nasal dorsum length.

After Holm–Bonferroni adjustment, 2 of 17 parameters remained significant in the CW group and 6 of 17 in the CCW group. Direct comparison between groups indicated that the overall extent of nasal soft-tissue changes did not differ significantly between CW and CCW rotation groups

## Data Availability

The data analyzed in this study includes photographs of individuals and is therefore not publicly available. Informed consent for the use of individual photographs was obtained from every participant depicted in this manuscript.

## Declarations

### **Clinical trial registration number**

DRKS (German Clinical Trials Register), DRKS00037763 (Registration Date: 25.08.2025).
